# Prises de risques chez les jeunes de Bobo Dioulasso: une analyse des facteurs associés à la précocité et au multipartenariat sexuel

**DOI:** 10.11604/pamj.2016.25.132.9767

**Published:** 2016-11-02

**Authors:** Clétus Come Adohinzin, Nicolas Meda, Adrien Marie Gaston Belem, Georges Anicet Ouédraogo, Issiaka Sombie, Abdramane Berthe, Laurence Fond-Harmant

**Affiliations:** 1 Organisation Ouest Africaine de la Santé (OOAS), Bobo-Dioulasso (Burkina Faso); 2 Université de Ouagadougou 03BP 7021 Ouagadougou 03 (Burkina Faso); 3 L'Université Polytechnique de Bobo-Dioulasso (Burkina Faso); 4 Centre Muraz, Bobo-Dioulasso (Burkina Faso); 5 Epidemiology and Public Health Research Unit, Luxembourg Institute of Health (Luxembourg)

**Keywords:** Sexualité précoce, comportements préventifs, multipartenariat, VIH/Sida, Burkina Faso, Sexual precocity, preventive behaviors, multiple partners, HIV/AIDS, Burkina Faso

## Abstract

**Introduction:**

Malgré les efforts d'éducation à la santé, les jeunes continuent d'adopter des comportements sexuels à risques, susceptibles d'avoir des répercussions importantes sur leur santé. Cette étude visait à analyser les facteurs associés à la précocité sexuelle et au multipartenariat chez les jeunes de 19-24 ans de Bobo-Dioulasso.

**Méthodes:**

Il s'agit d'une étude quantitative et transversale. Les données d'enquête ont été recueillies en décembre 2014 à Bobo-Dioulasso (Burkina Faso), auprès de 573 jeunes de 15 à 24 ans. Ces enquêtés ont été sélectionnés par un sondage en grappes à deux degrés. Des facteurs à risques relatifs à la précocité sexuelle et au multipartenariat ont été analysés à l'aide du logiciel Stata IC 13. Le seuil de signification de P<0,05 a été utilisée.

**Résultats:**

Plus de la moitié des enquêtés (54%) étaient sexuellement actifs dont 14% avant l'âge de 16 ans. Le multipartenariat sexuel avait été observé chez 24% des jeunes sexuellement actifs. Parmi les facteurs déterminants de la précocité sexuelle et du multipartenariat figuraient l'âge, le sexe, le niveau d'étude, et la situation économique des parents. Nos données avaient aussi montré que les rapports sexuels trop précoces étaient associés au multipartenariat sexuel (p<0,005).

**Conclusion:**

Les actions visant à renforcer les capacités des jeunes à retarder les premiers rapports sexuels et à mieux évaluer les risques seront de toute importance. Les capacités des parents, des enseignants et des prestataires devraient être aussi renforcées pour l'amélioration de la qualité des relations entre eux et les jeunes.

## Abstract (en)

**Introduction:**

Despite health education efforts, young people continue to adopt risky sexual behaviors which may have a significant impact on their health. This study aims to analyze the factors associated with sexual precocity and multiple partners among young people aged 19-24 years living in Bobo-Dioulasso.

**Methods:**

We conducted a quantitative, cross-sectional study. Survey data were collected from 573 young people aged 15-24 years in Bobo-Dioulasso (Burkina Faso) in December 2014. The respondents were selected using two stages cluster sampling. Risk factors for sexual precocity and multiple partners were analyzed using Stata 13 IC software. We used P<0.05 as the significance level.

**Results:**

More than half of respondents (54%) were sexually active, of whom 14% before the age of 16. Having multiple sex partners was reported by 24% of sexually active youth. Determinants of sexual precocity and multiple partners were age, sex, level of education and economic status of parents. Our data have also shown that early sexual debut was associated with multiple sexual partners (p<0.005).

**Conclusion:**

Actions aimed to convince young people to delay sexual debut and to better assess risks are of utmost importance. Enhancing parents, teachers and carers capabilities is essential to improve the quality of their relationships with young people.

## Introduction

Selon l'OMS, l'adolescence est une période de préparation à l'âge adulte au cours de laquelle ont lieu des étapes clés du développement. C'est souvent à ce moment que les individus deviennent sexuellement actifs, et acquièrent des compétences essentielles sur leur santé sexuelle et reproductive. Tout dépend donc de comment cette période transitoire est négociée. Si elle est mal négociée, ces étapes peuvent comporter des risques pour la santé à court ou à long terme [1]. Au Burkina Faso, de nombreuses filles se marient et donnent naissance à un enfant durant l'adolescence. Bon nombre de garçons débutent aussi leur vie sexuelle à cette période et se marient plus tard. D'autres doivent faire face à des relations sexuelles forcées, au mariage précoce, aux infections sexuellement transmissibles dont le Sida, ou encore aux grossesses non désirées et aux avortements clandestins [2]. Il est donc primordial que les jeunes puissent être accompagnés efficacement par les familles et les communautés, car ils sont plus susceptibles d'adopter des comportements protecteurs pour eux-mêmes et pour autrui, lorsqu'ils disposent d'une information appropriée sur leur santé sexuelle et reproductive. Plusieurs recherches se sont intéressées à la sexualité des jeunes. Les résultats de ces travaux ont montré qu'au Burkina Faso comme dans d'autres pays africains, une persistance des comportements sexuels à risques tels que la précocité des premiers rapports sexuels, la multiplicité des partenaires sexuels, les échanges économico-sexuels, la faible utilisation du préservatif et des contraceptifs [2–4]. C'est dans ce contexte que notre recherche s´est intéressée aux facteurs associés à la précocité des premiers rapports sexuels, et à la multiplicité des partenaires sexuels. Elle s´avère d´autant plus pertinente que, d'une part, les quelques études effectuées sur la précocité sexuelle et le multipartenariat à Bobo Dioulasso ne sont pas des plus récentes et, d'autre part, à cause du lien de « cause à effet » qui existe entre ces deux comportements, et qui sont documentés par certains auteurs. Il apparaît par exemple, que les rapports sexuels précoces augmentent le risque de transmission des IST et du VIH, et exposent au multipartenariat sexuel qui augmente également le risque de transmission du VIH [3]. Dans les sociétés burkinabè qui connaissent des mutations dues à plusieurs réalités, notamment l'urbanisation, l'analphabétisme, les inégalités liées au genre, et plus récemment les crises socio-économiques, les facteurs associés aux comportements sexuels à risques, requièrent donc une analyse rigoureuse. Cette étude visait à identifier les facteurs qui expliquent mieux la précocité sexuelle et le multipartenariat chez les jeunes de 19-24 ans de Bobo Dioulasso, afin de mieux contribuer à la prévention des IST/VIH/Sida, et à la réduction des grossesses non désirées.

## Méthodes

### Type d'étude, population cible et échantillonnage

Cet article traite des résultats d'une étude quantitative et transversale menée auprès de 573 jeunes âgés de 15 à 24 ans, résidant dans la commune urbaine de Bobo-Dioulasso au Burkina Faso. Tous les jeunes qui ne souhaitaient pas participer à l´enquête, ou qui ne répondaient pas aux critères d'âge et de résidence avaient été exclus. Deux comportements sexuels à risques, ont été analysés à travers cet article : la précocité sexuelle et le multipartenariat. La précocité sexuelle est définie comme étant le fait d'avoir sa première relation sexuelle avant l'âge de 16 ans. Le multipartenariat renvoie à l'existence, pour un individu donné à un moment donné, de plusieurs partenaires sexuels (simultanément ou successivement). Selon le Recensement Général de la Population et de l'Habitation [5], la commune urbaine de Bobo-Dioulasso comptait 489 967 habitants répartis dans 94 947 ménages, dont 24,95% de jeunes de 15-24 ans. Sur cette base, nous avons utilisé la formule de Machin [6] pour obtenir un échantillon de 573 individus. Le niveau de confiance était fixé à 95%, le taux de non-réponse à 10%, la précision ou marge d'erreur a été fixée à 5% (d=0,05).

### Collecte de données

Les jeunes ont été interrogés du 15 décembre 2014 au 22 janvier 2015. Les entretiens ont été conduits à domicile par 13 enquêteurs (enquêteurs et superviseurs). A la fin de chaque journée d'enquête, une réunion était organisée pour évaluer la cohérence des informations recueillies, et la complétude des questionnaires. Deux types de questionnaires (ménage et individuel) ont été utilisés, après leur validation par le comité éthique du Centre Muraz. Ils ont été élaborés sur la base de modèles de questionnaires utilisés dans le cadre des Enquêtes Démographique et de Santé du Burkina Faso [2]. Ils ont été accompagnées d'un formulaire de consentement éclairé, qui a été signé et daté par chaque enquêté. La collecte de données a été précédée par une formation des enquêteurs et des superviseurs sur l'éthique et la recherche quantitative et qualitative. Ces enquêteurs ont été sélectionnés sur la base de leurs expériences du travail d'enquêtes de terrain, et leur maîtrise de la langue locale. A l'issue de cinq jours de formation, un pré test a été organisé sur un site neutre qui ne fait pas partie de la zone d'enquête. Un atelier de validation des deux outils de collecte de données a été enfin organisé.

### Analyse des données

L'analyse statistique a été réalisée à l'aide du logiciel Stata IC 13, après une double saisie et le nettoyage de la base de données. Les données recueillies ont bénéficié d'un tri à plat et de calculs de mesures d'associations. Les statistiques descriptives des premières expériences sexuelles des jeunes, et la dynamique des relations sexuelles (multipartenariat) ont été présentés, stratifiées par tranche d'âge, par sexe, par niveau d'étude, par situation économique des parents et par utilisation du préservatif. Ces variables ont été comparées à l'aide des tests Chi-2 et le test exact de Fisher dépendant de l'effectif (Chi-2 pour les effectifs > 5 et test exact de Fisher ≤ 5). Une régression logistique a été menée, afin d'identifier les facteurs associés aux comportements sexuels à risques chez les enquêtés. Les valeurs p<0,05 étaient considérées comme statistiquement significatives.

## Résultats

### Caractéristique de l'échantillon par sexe

Le Tableau 1 montre que les jeunes interrogés étaient majoritairement des filles (53,6%). En considérant l'ensemble de l'échantillon, 91,6% d'entre eux étaient scolarisés tandis que 8% n'avaient jamais fréquenté l'école. Il s'agit d'une population essentiellement célibataire, dont 86,1% cohabitaient avec au moins un parent biologique ou un tuteur. La répartition des jeunes selon le lien avec le Ch ef de Ménage (CM), montrait que 65,6% d'entre eux étaient apparentés au CM (enfants/neveux/nièces/petits-fils) contre 34,4% sans lien de parenté avec le CM (enfant placé et ou adopté). Sur le plan socioéconomique, 72,6% des CM travaillaient alors que 27,4% n'avaient pas d'activité.

**Table 1:** Répartition des enquêtés, en fonction des caractéristiques socio-démographiques

Caractéristiques	Masculin N /%	Féminin N/%	Total N/%
**Total**	**266 (46,4)**	**307 (53,6)**	**573 (100)**
**Age**			
15-19	145 (54,5)	188 (61,2)	333 (58,1)
20-24	121 (45,5)	119 (38,8)	240 (41,9)
**Religion**			
Musulman	208 (78,2)	221(70,0)	429 (74,9)
Chrétien	55 (20,7)	86 (28,0)	141 (24,6)
Autres religions	3 (1,1)	0(0,0)	3 (0,5)
**Niveau scolaire actuel**			
Sans éducation formelle	11 (4,1)	37 (12,1)	48 (8,4)
Primaire	22 (8,3)	33 (10,7)	55 (9,6)
Secondaire	191 (71,8)	197 (64,2)	388 (67,7)
Supérieur	42 (15,8)	40 (13,0)	82 (14,3)
**Situation de cohabitation**			
Seul	8 (3,0)	1 (0,3)	9 (1,6)
Avec les parents biologiques	162 (60,9)	170 (55,4)	332 (57,9)
Avec tuteurs	83 (31,2)	79 (25,7)	162 (28,2)
Avec partenaire	0 (0,0)	41 (13,3)	41 (7,3)
Autres	13 (4,9)	16 (5,3)	29 (5,0)
**Lien de parenté avec le CM**			
Apparenté au CM (Enfants/ Neveux/nièces /petits- fils)	161 (6,5)	215 (70,0)	376 (65,6)
Non apparenté au CM (Enfant placé ou adopté)	105 (39,5)	92 (30,0)	197 (34,4)
**Statut professionnel du Chef de ménage**			
Avec travail	179 (67,3)	237 (77,2)	416 (72,6)
Sans travail	87 (32,7)	70 (22,8)	157 (27,4)

### La précocité sexuelle chez les jeunes de 15 à 24 ans de Bobo-Dioulasso

Les résultats de notre étude montraient que 54,0% de jeunes avaient déclaré avoir déjà eu leur premier rapport sexuel pénétratif. L'âge médian au moment des premiers rapports sexuels était de 17,75 ans pour les deux sexes (17,6 ans pour les filles et de 18 ans pour les garçons). On avait noté que 13,8% des jeunes avaient eu leur premier rapport avant 16 ans. Certaines caractéristiques de la précocité sexuelle chez les jeunes interrogés, sont résumées dans le Tableau 2. On peut constater à la lumière des résultats obtenus, une forte relation entre l'activité sexuelle avant l'âge de 16 ans, et certaines variables telles que la fréquentation scolaire, le sexe et l'âge. La fréquence des rapports sexuels précoces était plus élevée chez les jeunes non scolarisés que chez les jeunes scolarisés. Plus de la moitié des jeunes non scolarisés (62,8%) avaient eu leur premier rapport sexuel avant l'âge de 16 ans, contre 34,9% de ceux qui avaient un niveau correspondant au secondaire. C'est seulement 2,3% des jeunes du niveau supérieur qui avaient connu une entrée sexuelle précoce (p<0,001). Nous avons aussi noté que 41,9% des enquêtés avaient le premier rapport avec un partenaire de plus de 10 ans de différence, 23,2% avec un partenaire de 5 à 9 ans de différence d'âge, et 18,6% avec un partenaire de 1 à 4 ans de différence d'âge. C'est seulement 2,3% des jeunes filles qui avaient déclaré avoir eu le premier rapport avec un partenaire moins âgé qu'elles, et 13,9 % avec des partenaires du même âge. Ces différentes associations observées lors de l'analyse univariée entre la précocité sexuelle et les variables telles que le niveau scolaire, le sexe et l'âge, demeurent significatives dans les modèles multivariés.

**Table 2:** Répartition des enquêtés ayant déjà eu des rapports sexuels avant d’atteindre l’âge de 16 ans, en fonction des caractéristiques socio-démographiques

Caractéristiques	Premier rapport sexuel avant 16 ans (N/%)
**Total**	**43 (13,7)**
**Sexe**	
Masculin	16 (37,2)
Féminin	27 (62,8)
**P value**	**0,454**
**Niveau scolaire actuel**	
Sans éducation	27 (62,8)
Primaire	0 (0,0)
Secondaire	15 (34,9)
Supérieur	1 (2,3)
**P value**	**0,000**
**Ecart d'âge entre la fille et le premier partenaire**	
Moins âgé	1(2,3)
Environ même âge	6 (13,9)
1-4 ans de différence	8 (18,6)
5-9 ans de différence	10 (23,2)
plus 10 ans de différence	18 (41,9)
**P value**	**0,005**
**Utilisation de préservatif au premier rapport sexuel**	
Oui	20 (46,5)
Non	23 (53,5)
**P value**	**0,440**
**Utilisation de la contraception au premier rapport sexuel**	
Oui	21 (48,9)
Non	22 (51,1)
**P value**	**0,290**
**Situation économique du CM**	
Avec travail	32 (74,4)
Sans travail	11 (25,6)
**P value**	**0,287**

### Le multipartenariat sexuel chez les jeunes de 15 à 24 ans de Bobo-Dioulasso

Il ressort des données observées, que 24,4% des jeunes sexuellement actifs, avaient au moins deux partenaires sexuels différents au cours des 12 mois qui avaient précédé notre enquête. Parmi ceux-ci, 61,8% étaient des garçons et 38,2% des filles. La classification du type de multipartenariat avait montré que 66,4% des jeunes concernés par le multipartenariat, avaient des rapports sexuels concurrentiels (concomitants), contre 33,6% des jeunes qui entretenaient des rapports sexuels successifs avec plus d'un partenaire pendant cette période (sériels). Plusieurs raisons ont été évoquées par les enquêtés pour justifier le multipartenariat : 65,9% pour la recherche d'un ou d'une partenaire sûr (e), 18,2% en échange d'argent ou de cadeaux, et 15,9% pour des raisons émotionnelles. Nos résultats suggéraient une forte relation entre le multipartenariat et le sexe, l'usage du préservatif et la situation économique du ménage. Tous autres facteurs demeurant égaux, le multipartenariat (simultanément ou successivement) était plus fréquemment rapporté par les garçons que par les filles (61,8% contre 38,2% ; p<0,001). De la même façon, on avait constaté que les multipartenaires étaient les plus nombreux à avoir déclaré l'usage du préservatif au cours des douze derniers mois (p<0,001). Les jeunes enquêtés qui changeaient souvent de partenaires, se protégeaient plus (88,2%) que ceux qui avaient eu peu de partenaires (11,8%). La situation économique du Chef de Ménage était fortement associée au multipartenariat (p=0,002). Selon le Tableau 3, le multipartenariat était plus fréquemment rapporté par les jeunes vivants dans les ménages où les chefs n'occupaient pas d'emploi (69,7%), que dans ceux où ils avaient un travail (30,3%). Par ailleurs, les analyses multivariées effectuées, avaient révélé une corrélation entre le multipartenariat et la précocité sexuelle. Ainsi, 70,4% des jeunes qui avaient eu leur premier rapport sexuel avant l'âge de 16 ans, avaient déclaré plusieurs partenaires, contre seulement 21,1% de ceux qui avaient déclaré une sexualité tardive (p=0,0341).

**Table 3:** Répartition des enquêtés ayant au moins deux partenaires sexuels (relation avec ou sans pénétration) au cours des 12 mois, en fonction des caractéristiques socio-démographiques

Caractéristiques	Partenaires multiples lors des 12 derniers mois (N/%)
**Total**	**76 (24,4)**
**Sexe**	
Masculin	64 (84,2)
Féminin	12 (15,8)
**P value**	**0.000**
**Age**	
15-19	24 (31,6)
20-24	52 (68,4)
**P value**	**0,168**
**Niveau scolaire actuel**	
Sans éducation	35 (46,0)
Primaire	0 (00)
Secondaire	32 (42,1)
Supérieur	9 (11,8)
**P value**	**0.343**
**Utilisation de préservatif avec tous les partenaires**	
Oui	67 (88,2)
Non	9 (11,8)
**P value**	**0,000**
**Situation économique du CM**	
Chômeur	53 (69,7)
Travailleur	23 (30,3)
**P value**	**0,047**

## Discussion

Cette étude avait comme principal objectif d'analyser les facteurs associés à la précocité sexuelle et au multipartenariat chez les jeunes de 19-24 ans de Bobo-Dioulasso. Les données de cette enquête montrent une persistance des comportements sexuels à risques tels que la précocité des premiers rapports sexuels et le multipartenariat. Cette partie nous permettra d´interpréter ces différents résultats de notre recherche.

### Les déterminants d´une entrée précoce dans la vie sexuelle

A l'image d'autres recherches menées au Burkina Faso et ailleurs, cette étude a mis en évidence l'entrée en activité sexuelle précoce des jeunes. Plus de la moitié des enquêtés avaient déjà eu leur premier rapport sexuel, dont 13,7% avant l'âge de 15 ans. En 2004, une étude menée au Burkina Faso par Gueilla [7] avait noté que l'âge médian lors du premier rapport sexuel, était de 17,2 ans pour les femmes et de 19,7 ans pour les hommes. Un peu plus tard en 2010, EDS-IV [2] avait relevé un rajeunissement de l´âge médian au premier rapport sexuel uniquement chez les jeunes filles. Il était passé de 17, 2 ans en 2004 à 17,7 ans en 2010. Ces constats sont assez préoccupants, vu l´âge médian auquel ces jeunes avaient eu leur premier rapport sexuel pénétratif. A cet âge, ils ne sont pas encore matures. Une explication de la précocité sexuelle peut en être que, les jeunes grandissent dans un environnement rapidement changeant, qui leur offre continuellement de nouvelles découvertes. La mondialisation, l'accès à de nouvelles techniques de communication, l'urbanisation rapide et l'évolution des normes sociales, sont tous des facteurs qui attisent la curiosité des jeunes. D'autres explications ont été suggérées par de nombreuses recherches, pour justifier le rajeunissement de l´âge médian au premier rapport sexuel. Pour Adjahoto [8], cette situation est la conséquence du déficit de communication entre les jeunes et leurs parents. Cette difficulté de communication amène les enfants à une recherche d'informations parfois non fiables en dehors du cercle familial (dans la rue ou avec des amis). Il nous paraît important de reconnaître l'évolution des réalités de la vie des adolescents et des jeunes. Une éducation sexuelle complète de bonne qualité, offre un espace pour ce dialogue dans les milieux éducatifs, les centres de santé, entre pairs, au sein de la famille et dans la communauté [9].

La grande majorité de nos enquêtés avaient leur premier rapport avec un partenaire plus âgé. Cette observation est en conformité avec la préférence clairement affichée par les jeunes de Bobo-Dioulasso, lors d'une étude précédente menée par Baya [10]. Les résultats de cette étude indiquaient un écart de 6,5 ans entre les premiers partenaires sexuels. Par ailleurs, le fait d'avoir un premier partenaire plus âgé, était associé à des premiers rapports sexuels précoces chez les jeunes. Cette association justifie le fait que les premières expériences sexuelles, soient généralement vécues avec des personnes expérimentées. Selon Bozon [3], 83% des partenaires des femmes ont plus d'expériences sexuelles qu'elles. De notre point de vue, ce déséquilibre d'expériences peut avoir des conséquences importantes sur la santé du partenaire le plus jeune (violences et abus sexuels, infections aux IST/VIH et grossesses non désirées). En 2003, une étude menée aux Etats-Unis [11] avait justifié le taux élevé de la prévalence du VIH chez les jeunes femmes, par la différence d'âge entre celles-ci et leurs partenaires sexuels. Du reste, l'écart d'âge est un indicateur dont il faudra tenir compte dans l'élaboration des programmes de prévention. Nos résultats ont aussi révélé une corrélation positive entre la fréquentation scolaire et la précocité sexuelle. On peut estimer que l'environnement éducatif, crée des espaces et des occasions d'échanges d'informations, permettant aux jeunes de prendre de bonnes décisions en ce qui concerne leur sexualité. Selon l'UNICEF [12], les jeunes instruits sont susceptibles d'adopter des attitudes et d'avoir des connaissances qui leur permettent de résister aux pressions, et d'assumer la responsabilité de leur propre vie. Pour que cet avantage profite mieux aux jeunes, les informations sur la sexualité ainsi que sur les compétences essentielles, devraient être intégrées au niveau primaire et offertes pendant toute la scolarité. Les jeunes qui ne resteront pas très longtemps dans le système scolaire pourront ainsi en bénéficier.

### Le multipartenariat et les facteurs associés

Le multipartenariat sexuel est une pratique déclarée par 24% des jeunes enquêtés, bien que cette pratique semble accroître le risque d'exposition au VIH/Sida et aux grossesses non désirées. La fréquence du multipartenariat parmi les jeunes, avait été déjà documentée par plusieurs études au Burkina Faso. L'EDS IV [2] avait révélé que 11,5% des jeunes garçons de 15 à 24 ans, avaient eu des rapports sexuels avec au moins deux partenaires, contre 0,9% pour les jeunes filles. Par ailleurs, nous avons noté que le multipartenariat concomitant était plus fréquent (66,4%) chez les enquêtés que le multipartenariat sériel. Pourtant, plusieurs recherches avaient montré que le multipartenariat concomitant demeure l'un des risques majeurs, conduisant l'épidémie du VIH en Afrique [7, 13]. Pour Gueilla [7], les rapports sexuels concurrentiels sont en effet très déterminants dans la propagation du VIH, notamment dans des situations où le condom n'est pas utilisé. Cependant, d'autres études avaient montré que le multipartenariat ne serait pas si déterminant dans la transmission des VIH/Sida et les grossesses non désirées. Selon Ferry [14], le nombre de partenaires sexuels n'influencerait pas de façon importante sur le taux de transmission du virus dans une population sexuellement active. Mais il serait un facteur accroissant le risque de transmission en présence d'autres facteurs (rapport anal, absence de circoncision, non usage de préservatif). Pour Bajos [15], la prise de risques peut être moins importante chez un multipartenaire qui protège chaque rapport sexuel, que chez un monopartenaire qui a des rapports non protégés.

Les résultats de notre étude ont montré que le multipartenariat sexuel est, quel que soit l'âge, plus intense parmi les garçons que les filles. Cette différence témoigne des rapports inégaux entre les hommes et les femmes. Elle se caractérise par des stéréotypes sexuels (masculins et féminins) produits par la société. Pour Bergeron [13], la tradition et les représentations sociales ont favorisé une conception bipolaire des rôles sexués. L'homme doit être autonome, actif et compétitif. En revanche, l'identité féminine se définit en fonction d'autrui et dans des qualificatifs tels que l'affectivité, la passivité et la vulnérabilité. On peut estimer que, ce sont ces attentes sociales sur la façon dont les hommes doivent se comporter, qui parfois amènent les jeunes à adopter des comportements à risques tel que le multipartenariat sexuel. Dans une étude réalisée dans les pays francophones de l'Afrique subsaharienne y compris le Burkina Faso, Rwenge [16] avait montré que les sociétés africaines étaient plus bienveillantes à la sexualité des hommes, par rapport à celle des femmes. Elles n'attendent pas la même chose des garçons et filles dans l'activité sexuelle. Les garçons doivent être sexuellement expérimentés avant de se marier, alors que les filles doivent rester vierges. Notre étude avait établi une association positive entre le multipartenariat et l'utilisation du préservatif. Les jeunes qui changeaient ou multipliaient les partenaires, se protégeaient plus (88,2%) que ceux qui avaient eu peu de partenaires (11,8%). Cela pourrait s'expliquer par le fait que les premiers sont conscients des risques liés au multipartenariat (infections aux VIH/Sida et les grossesses non désirées). Selon Dab [17], ce sont la perception du risque et la proximité subjective avec la maladie, qui justifient l´utilisation du préservatif chez les multipartenaires. Bien que le constat soit encourageant, nous ne pouvons malheureusement pas oublier que l'efficacité du préservatif est déterminée par son utilisation correcte. Ainsi, pour Ferry [14], ce n'est pas le nombre ou le pourcentage d'utilisation qui est le plus important, mais les conditions d'utilisation du préservatif. Cela devrait inciter au renforcement des campagnes de sensibilisation plus diversifiées, pouvant améliorer les compétences des jeunes pour une prévention plus efficace. Comme chez Rwenge [16], nos résultats avaient montré que les jeunes dont les parents étaient sans emploi, qui vivaient dans des foyers pauvres, présentaient un plus grand risque de partenaires multiples. Ceci pourrait trouver son explication dans le modèle de comportement sexuel fondé sur la théorie dite «économique ». Cette théorie considère un rapport sexuel, comme étant un acte bien réfléchi et motivé par un intérêt économique (matériel ou financier). Les jeunes filles ont des rapports sexuels avec les hommes aisés, plus âgés et déjà mariés, afin de profiter de certains privilèges, et cela parfois, avec la bénédiction tacite ou sous la pression soutenue des parents eux-mêmes, désireux de voir partir rapidement leur fille, et alléger quelque peu la charge financière de leur ménage [18, 19]. D'autres auteurs avaient nuancé les causes économiques du multipartenariat [20, 21]. Pour eux, il faut faire la différence entre la jeune fille qui choisit de commencer une activité sexuelle et de recevoir par la suite des cadeaux, et celle qui s'y engage pour des raisons économiques. Car, recevoir de l'argent ou des cadeaux d'un homme avec qui on a des rapports sexuels, peut être normal dans certaines cultures [21].

### La précocité sexuelle et le nombre ultérieur de partenaires

Il ressort de nos données, que les jeunes ayant commencé leur vie sexuelle de façon précoce, étaient plus enclins au multipartenariat sexuel que ceux qui avaient une sexualité tardive. On peut en déduire que les individus plus précoces sexuellement, connaissent une vie sexuelle plus longue, une plus grande diversité d'expériences et de pratiques sexuelles. C'est aussi possible qu'une entrée précoce dans la sexualité retarde la maturation sociale, ce qui influencerait le comportement sexuel futur des jeunes. De tels résultats avaient été déjà obtenus par plusieurs études en Afrique et ailleurs. Au Kenya, Akwara [22] avait constaté que la précocité sexuelle contribue à la vulnérabilité au VIH, à travers l'exposition des adolescents à un plus grand nombre de partenaires sexuels, et à une plus longue période d'activité sexuelle avant l'établissement de relations monogames à long terme. Selon Bozon [3], le fait que certains individus connaissent un âge précoce ou tardif au premier rapport, préfigure une attitude durable à l'égard de la sexualité, et plus largement à l'égard du couple, voire de la vie familiale. Il est nécessaire que les familles aident les jeunes à retarder le plus possible, le premier rapport sexuel. Une éducation sexuelle de qualité favorisera des comportements à moindre risque. Idéalement, les parents devraient assumer ce rôle, puis l'école, et la communauté. Cependant, de récentes statistiques attestent que les jeunes s'instruisent plutôt auprès de sources ambivalentes telles que les amis et la télévision [23].

Notre étude présente plusieurs limites. La première est relative à l´aspect rétrospectif de nos deux principales variables : l'âge aux premiers rapports sexuels et le nombre de partenaires sexuels au cours des douze derniers mois précédant l'enquête. Des erreurs de mémoire sont possibles, surtout pour les jeunes dont les premiers rapports ont eu lieu longtemps avant notre enquête. En deuxième lieu, le caractère tabou de la sexualité est un aspect pouvant influencer la qualité des réponses. Certains jeunes peuvent volontairement fournir des fausses réponses pour protéger leur vie privée, par crainte d'une censure ou pour se vanter. Néanmoins, les informations obtenues se révèlent convergentes aux conclusions de certaines études antérieures, preuve que ces limites ne sont pas de nature à jeter de doute sur la pertinence de cette recherche.

## Conclusion

Malgré leurs bonnes connaissances dans le domaine de la prévention du VIH/Sida, les jeunes continuent d´adopter des comportements qui les exposent au VIH/Sida et aux grossesses non désirées. Ces comportements à risques sont déterminés par les facteurs individuels (l'âge et le sexe), socio-culturels (le niveau scolaire et les normes sociales et culturelles) et environnementaux (la modernisation des valeurs et la situation économique). Il est urgent de réduire la vulnérabilité sexuelle des jeunes. Les actions concertées de renforcement des capacités des jeunes à adopter des comportements à moindre risque, sont de la toute première importance. Ces actions doivent être menées avant le début de l'activité sexuelle, au sein de la famille, à école, sur les lieux d'apprentissage, dans les centres de santé, et aux centres de loisirs des jeunes [4, 9]. Les capacités des parents, des prestataires de services et des éducateurs, doivent être aussi renforcées pour l'amélioration de la qualité de la communication entre eux et les jeunes.

### Etat des connaissances actuelles sur le sujet

- C'est souvent lors de l'adolescence que les individus deviennent sexuellement actifs, et acquièrent des compétences essentielles sur leur santé sexuelle et reproductive. La sexualité future des adolescents et jeunes dépend de comment cette période transitoire est négociée;
- Les comportements sexuels à risques sont multiples et varient d'un jeune à un autre, et d'un milieu culturel à un autre. Ils renvoient aux environnements sociaux, psychosociaux, financiers ou matériels.

### Contribution de notre étude à la connaissance

- La précocité sexuelle et le multipartenariat sont des pratiques sexuelles vécues par une frange non négligeable de notre population d'étude;
- Notre étude met en lumière l'importance d'une pratique sexuelle plus tardive dans la réduction du multipartenariat sexuel;
- Elle suggère des interventions préventives précoces dans une approche globale, qui vise non seulement la transmission de connaissances, mais surtout d'encourager d'adoption de comportements pouvant permettre aux jeunes de retarder l'échéance du premier rapport sexuel, et de mieux évaluer les risques sexuels.

## References

[1] Organisation Mondiale de la Santé (OMS) Risques pour la santé des jeunes. 64ème Assemblée mondiale de la Santé..

[2] Institut national de la statistique et de la démographie (2010). Enquête démographique et de santé et à indicateurs multiples (EDSBF -MICS IV) du Burkina Faso..

[3] Bozon M, Kontula O (1997). Initiation sexuelle et genre: comparaison des évolutions de douze pays européens. Population..

[4] Berthé A, Huygens P, Ouattara C, Sanon A, Ouédraogo A, Nagot N (2008). Comprendre et atteindre les jeunes travailleuses du sexe clandestines du Burkina Faso pour une meilleure riposte au VIH. Santé..

[5] Institut national de la statistique et de la démographie (INSD) (2009). Analyse des résultats du recensement général de la population et de l'habitat..

[6] Machin D, Campbell J, Tan S, Tan S (2009). Sample Size Tables for Clinical Studies..

[7] Guiella G (2004). Santé Sexuelle et de la Reproduction des Jeunes au Burkina Faso:Un Etat des Lieux..

[8] Adjahoto, Hodonou K, De Souza A, Tété V, Baeta S (2000). Information des jeunes en matière de sexualité. Cahiers d'études et de recherches francophones..

[9] United Nations Educational, Scientific and Cultural Organization (UNESCO) (2013). Young People Today. Time to Act Now..

[10] Baya B (2004). Fréquentation scolaire des jeunes filles et risques d'infection à VIH: espoir ou inquiétude? Le cas de Bobo-Dioulasso au Burkina Faso. Etude de la population africaine..

[11] DiClemente RJ, Crosby RA, Wingood GM (2002). Comment prévenir l'apparition du VIH chez les adolescents: une ébauche de solutions pour combler les lacunes. Perspectives..

[12] Fonds des Nations Unies pour l'Enfance (UNICEF) (2011). La situation des enfants dans le monde..

[13] Bergeron A, Gaudreau L (1985). Identité sexuelle et intervention en sexualité humaine. Cahiers Sexol..

[14] Ferry B, Charles Becker, Dozon JP, Obbo C, Touré M (1999). Systèmes d'échanges sexuels et transmission du VIH/sida dans le contexte africain. Vivre et penser le sida en Afrique. Experiencing and understanding AIDS in Africa..

[15] Bajos N, Maia M, Bozon M, Ferrand A (1999). La sexualité au temps du sida. L'Homme..

[16] Rwenge J (2013). Comportements sexuels parmi les adolescents et jeunes en Afrique Subsaharienne Francophone et facteurs associés. African journal of reproductive health..

[17] Dab W, Moatti JP, Beltzer N (1993). Les modèles d'analyse des comportements à risque face à l'infection à VIH: une conception trop étroite de la rationalité. Population..

[18] Görgen R, Katzan J (1995). La sexualité des jeunes, résultats d'une étude qualitative sur les attitudes, les croyances et comportements des jeunes de Bangui..

[19] Calvès AE (1993). Youth and Fertility in Cameroon: changing patterns of family formation. (Thesis of Rural Sociology and Demography)..

[20] Tantchou JC (2009). Santé reproductive des jeunes: une revue critique de la littérature. Natures Sciences Sociétés..

[21] Nyanzi S, Pool R, Kinsman J (2001). The negotiation of sexual relationships among school pupils in south-western Uganda. AIDS Care..

[22] Akwara PA, Madise NJ, Hinde A (2003). Perception of risk of HIV/AIDS and sexual behaviour in Kenya. Journal of Biosocial Science..

[23] Yode ML, LeGrand T (2012). Association between age at first sexual relation and some indicators of sexual behaviour among adolescents. Afr J Reprod Health..

